# How to figure out what CPD/CME you need

**Published:** 2017-05-12

**Authors:** Milka Mafwiri, Nick Astbury

**Affiliations:** 1 Senior lecturer and Consultant Ophthalmologist, and former Associate Dean for undergraduate studies of the School of Medicine, Muhimbili University of Health and Allied Sciences, Dar es Salaam, Tanzania.; 2 Clinical Senior Lecturer: International Centre for Eye Health, London School of Hygiene and Tropical Medicine, London, UK.

**Do you feel totally in control of every situation? We need to be honest about the gaps in our knowledge or skills, keep motivated and find solutions to the challenges.**

**Figure:**
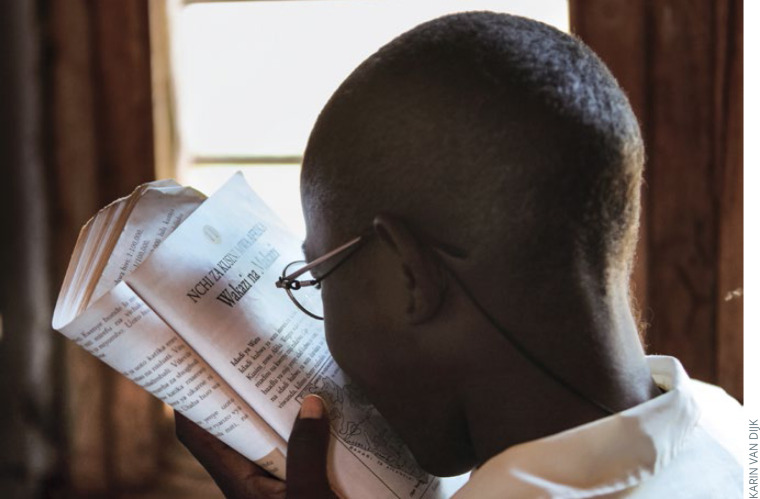
How much do you know about low-vision and low-vision aids? TANZANIA

## Identifying the need

Clinicians are usually very busy with their clinical practice and may not have thought about their needs relating to CME and CPD. However, effective CPD requires prior preparation. Every clinician may experience a feeling of unease or lack of confidence when faced with a difficult situation. The underlying cause may be a lack of knowledge or skill, which if remedied would enhance the outcome for the patient and the job satisfaction for the clinician.

Identifying the need requires being honest with oneself and realising that everyone, whether junior or senior members of staff, has the potential and an obligation to maintain standards of practice and patient care (see article on page 13 for various reasons for keeping up to date).

Different types of CPD activities may be chosen according to the identified needs. The following table is an example of how a clinician can identify what CPD activity may be relevant for them.

**Table 1:** Identifying relevant CPD activities

Knowledge gap	Skills updating	Acquisition of competency	Performance demonstration
**Failed treatment** of a certain condition. Need to find out if there has been a change in the treatment of the condition and factors associated with recurrence e.g. squamous cell carcinoma of the conjunctiva.	**Noting lack of skill in using certain equipment.** Need to understand or refresh techniques e.g. applanation tonometry, indirect ophthalmoscopy, use of ophthalmic (BIO) lenses.	**Lack of confidence in treatment** of certain conditions. Practice required.	How to teach others on the subject of interest. Sharing and dissemination to peers through publication.
**Inability to diagnose** a clinical condition by matching the symptoms and signs. Need to work out the differential diagnosis by accessing information.	**Noting deficiency in performance** of a surgical procedure: e.g. cataract surgery (small incision or phacoemulsification).		
**Inadequate up-to-date information** on certain conditions common in your area. Need clinical guidance. e.g. classification and treatment of diabetic retinopathy or retinoblastoma.	**Noting inability to perform or interpret a procedure after acquiring a new diagnostic machine** e.g. A scan biometer, Optical Coherence Tomography (OCT), iCare tonometer, visual field machine.	**Lack of confidence in performance** of certain steps in a surgical procedure. Practice required.	How to share what you are doing with others. Listening, learning and sharing your knowledge with others to improve your performance.
**Inadequate knowledge** about a common condition in your area that was not learnt during basic training e.g. low vision and low visual aids.	**Learning a new surgical skill** after getting new equipment e.g. learning to perform phacoemulsification with a new machine.		

**Figure:**
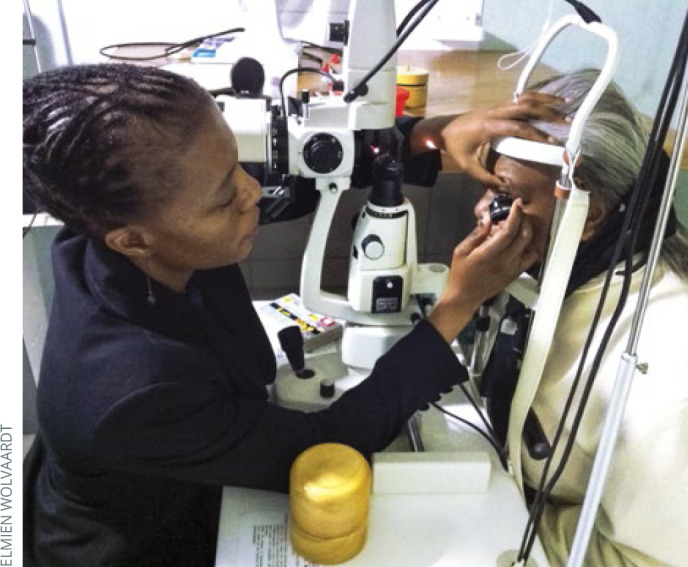
Do you feel competent treating a patient with diabetic retinopathy? SOUTH AFRICA

## Motivation

It is necessary to be motivated to attend CME/CPD training. Motivating factors broadly fall into ‘carrot’ and ‘stick’ categories. It is best to be motivated by positive factors (carrots) that lead to fulfilment, increased self-esteem and good outcomes for your patients rather than feel that CPD is an unnecessary chore that has to be done to avoid a penalty (stick).

Some motivating factors are:

- A desire for delivery of high quality care and the best possible outcome for patients
- Recognition by patients and peers of the excellence of your work
- A desire to avoid ‘critical incidents’ or ‘near misses’ that are bad for patients and undermine confidence
- The need to satisfy requirements for registration and maintaining a licence to practise

**Figure:**
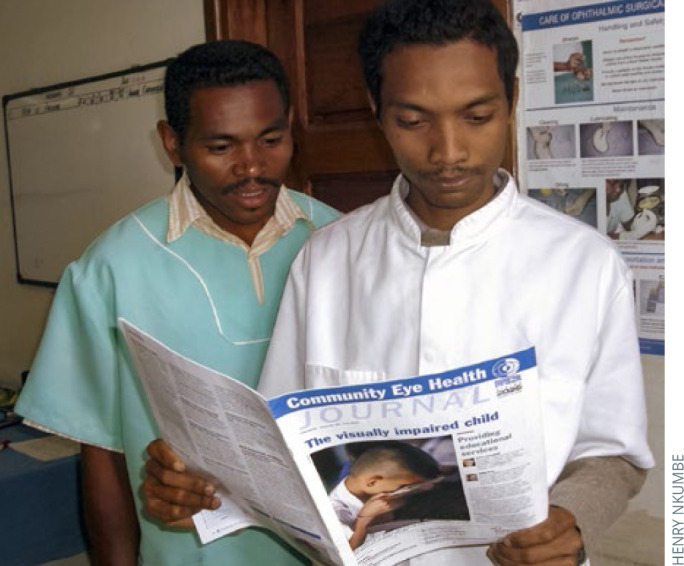
Make time to read the *Community Eye Health Journal*. MADAGASCAR

## Challenges

Busy clinicians face a number of challenges for attending CME/CPD. However keeping up to date is so important that every effort must be made to overcome the challenges and find solutions (see Table 2).

**Table 2:** Matching solutions to challenges

Challenges	Solutions
Identifying the need.	If things go wrong – keep a record to reflect on and identify gaps that can be filled by CPD. Ask colleagues how they keep up to date. Use appraisal with line managers, for advice on choosing relevant CPD activities.
Inadequate time: Most of the time clinicians are busy in their clinics from morning to evening especially in low resource settings.	Remember that keeping up to date is as important as seeing patients in the clinic or the operating theatre. Think about time management or take a course on time management. Set aside dedicated time to read (for example the *Community Eye Health Journal*). Keep a CPD diary and refer to it!
Because of their busy schedule they sometimes fail to get permission to attend external CPD/CME as they need to work. e.g. attending conferences.	Get organised. Plan ahead. Apply for study leave or funding in advance. Keep a diary.
Not knowing where to get CPM/CME especially for low resource settings.	Discuss with colleagues how they manage. Look for online courses. Many can be downloaded and used offline.
Lack of travel expenses to attend external CME/CPD - provision of CPDs at a central area within the country where many clinicians can attend.	Identify the most useful meetings, courses or events and opportunities for CPD and lobby your manager. CPD websites (e.g. COECSA).

